# Sleep at home for older persons with dementia and their caregivers: a qualitative study of their experiences and challenges

**DOI:** 10.1007/s10433-025-00858-w

**Published:** 2025-06-16

**Authors:** C. A. M. Huisman, E. R. C. M. Huisman, R. G. A. Brankaert, H. S. M. Kort

**Affiliations:** 1 Research Group Technology for Healthcare Innovations, Research Center Healthy and Sustainable Living, University of Applied Science Utrecht, Heidelberglaan 7, 3584CS Utrecht, The Netherlands; 2 Building Lighting Group, Eindhoven University of Technology, Eindhoven, The Netherlands; 3 Industrial Design, Eindhoven University of Technology, Eindhoven, The Netherlands; 4 Research Group Health Innovations & Technology, Fontys University of Applied Sciences, Eindhoven, The Netherlands; 5 Research Group Tranzo, Tilburg University, Tilburg, The Netherlands

**Keywords:** Alzheimer, Non-pharmacological interventions, Experience flow, Aging-in-place, Cognitive impairment, situation at night

## Abstract

Most people with dementia (PwD) live with the support of their caregiver. Sleep issues are common among all types of dementia and increase the burden on the IC. Disturbed nights may lead to earlier admission to nursing homes. This study explored the experiences and challenges related to the sleep of PwD and IC. A qualitative study using semi-structured interviews was performed. The target groups were PwD, ICs, and care professionals. The sample comprised 20 informal caregivers, 2 PwD, and 9 care professionals. Thematic analysis was used to identify patterns within and across data concerning the experiences and challenges of participants. In total, 31 participants were interviewed in 28 sessions. Three themes were identified, namely, (a) challenges in maintaining time orientation and day/night routines, (b) irregularities and concerns of informal caregivers at night, and (c) environmental cues that support or disturb sleep. The results provide insight into the experiences of IC at home regarding their sleep and the sleep of PwD at home. Our results may guide the development of non-pharmacological interventions to support sleep and day structure with a certain balance in activities.

## Introduction

The number of people over 65 years old is increasing, and as a result, the incidence of people with dementia (PwD) is also expected to increase. In 2015, 47 million people worldwide lived with dementia; this number is expected to reach 132 million by 2050 (World Health Organization 2017). Globally, dementia is a leading cause of dependency and disability among older people (Wortmann 2012). By 2050, the number of PwD in the Netherlands is expected to increase to 620,000 people (Alzheimer Nederland 2021). Dementia interferes with daily functioning, communication, and broader engagement in society, as it is a progressive syndrome that negatively affects memory, other cognitive abilities, and behavior (Winblad et al. 2016). Due to these challenges, PwD increasingly need support from (in) formal caregivers. The most common form of dementia is Alzheimer's disease and accounts for 75–80% of cases. Other forms include vascular dementia, dementia with Lewy bodies, and frontotemporal dementia (World Health Organization 2017).

The increasing number of PwD puts pressure on our care systems and increases healthcare costs. Additionally, many Western societies have a shortage of care staff and are unable to provide the care needed (World Health Organization 2022). Therefore, promoting aging-in-place (Verver et al. 2018), that is encouraging people to live at home as long as possible, is a key policy to improve the sustainability of care systems. This is also the preference of many PwD (Pani-Harreman et al. 2021). However, this approach shifts the demand and workload toward informal caregivers, which, in turn, dwindles alongside the workforce (Wieczorek et al. 2022). Research shows that providing informal care is stressful and hazardous to the health of informal caregivers (Roth et al. 2015). Informal caregiving can be emotionally and cognitively demanding, negatively affecting the overall well-being of informal caregivers (Chiao et al. 2015). Furthermore, it has been observed that cognitive functioning, verbal memory, and attention of informal caregivers are negatively affected compared to non-caregivers due to informal caregiving responsibilities (Brewster et al. 2022).

In the case of dementia, aging-in-place can be challenging as it increases the demands on their social support networks. The majority of individuals in the early to mid-stages of dementia globally receive support from an informal caregiver within their community (Greenwood and Smith 2019). Presently, in the Netherlands, 79% of the PwD are able to live at home with support from an informal caregiver (Alzheimer Nederland 2021). In some cases, they receive support from professional care, such as home care, case management, and daycare places.

An essential part of delivering effective care is making sure that caregivers get enough sleep themselves (Peng et al. 2019). Sleep quality can be defined as an individual's self-satisfaction with all aspects of the sleep experience. Sleep quality comprises sleep efficiency, latency, duration, and waking after sleep onset (Nelson et al. 2022). The sleep–wake rhythm changes with age (Gulia and Kumar 2018). In all types of dementia, sleep rhythms change. In the case of Alzheimer’s disease, there can be more significant sleep fragmentation, with more frequent, more extended periods of intra-sleep wakefulness (Bombois et al. 2010), which may lead to increased daytime irritability and decreased attention, motivation, and cognitive performance (Cipriani et al. 2015). PwD often deal with circadian and sleep disturbances compared with people of the same age without dementia. These disturbances negatively affect the quality of life and functional abilities of PwD and increase the care burden for caregivers (Ooms and Ju 2016). Approximately 60–70% of PwD have sleep disturbances (Petrovsky et al. 2018). Minimizing sleep disturbances may improve well-being, daytime functioning, and quality of life, and it may also slow the progression of dementia (Kinnunen et al. 2017). When sleep problems occur, they can also influence the informal caregiver. Sleep disturbances can exacerbate the changes in mental, physical, and cognitive health (Casagrande et al. 2022; Gothe et al. 2020). Research highlights that 50–70% of informal caregivers experience sleep disturbances (Byun et al. 2016). The sleep disturbances of PwD and the impact on the informal caregiver potentially lead to nursing home admission (Livingston et al. 2017; McCrae et al. 2016).

Therefore, exploring alternative and innovative ways to support sleep in PwD may relieve formal and informal caregivers, and thus support aging-in-place policy. To support aging-in-place policies, it is important to understand how the sleep of PwD can be improved. Besides that, the most promising interventions are multicomponent. Koren et al. (2023) suggested exploring the potential of interventions to support sleep to increase the time that PwD can live well at home and delay the transition to a long-term care facility. Phung et al. (2013) concluded that interventions should be customized to meet the specific needs of both PwD and the caregiver, potentially increasing their overall benefit. In this study, we explore the experiences regarding the sleep of PwD and their informal caregivers. The research question addressed is “What are the experiences, challenges, and wishes faced by PwD and their caregiver(s) regarding sleep?”

## Method

### Study design recruitment and population

We performed a qualitative study using semi-structured interviews to address the research question. The study was conducted in the Netherlands. In this study, we included individuals from migrant backgrounds to ensure inclusivity, as they are often excluded from research. Participants were recruited by the researchers and students, through various networks, as well as via health and welfare organizations. The recruitment was focused on caregivers (informal and formal) and on PwD. Formal caregivers (professionals) were recruited to capture their perspective on the experiences, challenges, and wishes faced by PwD and their caregiver(s) regarding sleep. Inclusion criteria were that participants with dementia could (1) participate in a conversation with the researcher, (2) informal caregivers needed to care for or have cared for someone with dementia, and (3) professionals (formal caregivers) must have worked with PwD. There were no explicit exclusion criteria.

The sample comprised 20 informal caregivers, 9 professional caregivers who were able to speak in favor of the PwD, and 2 PwD. While the participation of PwD is regarded as very important, our main outcomes are thus mainly based on the data gathered from formal and informal caregivers. There were 28 interviews performed, comprising 31 participants (three double interviews). The characteristics and numbers of participants are displayed in Table 1. Most participants consisted of informal caregivers caring for someone with dementia. In two cases, interviews were held with a person with dementia together with their caregiver.

**Table 1:** Overview of the period, topics, number of participants, and locations of the interviews performed. Period 1: informal caregivers; Period 2: informal caregivers/professionals; and Period 3: PwD and their caregivers or professionals

Topics	Participant(s)	N =	Interview location	Relation with PwD
Period 1: 1) sleep of PwD and informal caregivers, 2) day–night rhythm, 3) problems/struggles during day and night, 4) role of assistive technologies, and 5) activities during the daytime	Informal caregiver	11	Face-to-face (N = 2); Telephone (N = 9)	Partner (N = 9); Family member (N = 2)
Period 2: (1) sleep of PwD and informal caregivers, (2) day–night rhythm, (3) problems/struggles during day and night, (4) role of assistive technologies, and (5) activities during the daytime	Professional	7	Online (N = 7)	Professional (N = 7)
Period 3: (1) sleep of PwD and informal caregivers, (2) day–night rhythm, (3) problems/struggles during day and night, (4) role of assistive technologies, (5) activities during the daytime, (6) bedroom, and (7) nutrition	Informal caregiver with a person with dementia	2	Face-to-face (N = 2)	Partners (N = 2)
	Professional	2	Online (N = 2)	Professional (N = 2)
	Informal caregiver	2	Online (N = 1); Telephone (N = 1)	Partner (N = 1); Family member (N = 1)
Period 4 (1) sleep of PwD and informal caregivers, (2) day–night rhythm, (3) problems/struggles during day and night, (4) role of assistive technologies, (5) activities during the daytime, and (6) nutrition	Informal caregiver duo	1	Online (N = 1)	Family member (N = 2)
	Informal caregiver	3	Face-to-face (N = 2); Telephone (N = 1)	Partner (N = 2); Family member (N = 1)

Family members can be a son, daughter, or next of kin

### Ethics

The study followed the principles of the Declaration of Helsinki and the Code of Conduct for Health Research (World Medical Association 2014). The Health Domain Research Ethics Committee at Utrecht University of Applied Sciences approved the study protocol (ECO-GD-ref. 188-000-2022). The investigators’ protocol emphasizes conducting research in a respectful, honorable, and careful manner. Researchers confirm participants’ willingness to continue, allow them to stop the interview at any time, and ensure participants have either signed an informed consent form or given audio-recorded consent.

### Procedures

The interviews were held to retrieve the experiences, preferences of PwD and their caregivers during and around the night. The interviews were conducted in four periods (different regions and different target group) and by different interviewers (researchers and/or trained students), for each period, a topic list was developed to explore the following areas. The topic lists were iteratively developed based on the literature and advancing insights of the researchers based on the findings in the interviews. Topics included (1) the sleep of PwD and informal caregivers, (2) day–night rhythm, (3) problems/struggles during day and night, (4) role of assistive technologies, and (5) activities during daytime. The topics related to the bedroom (period 3) and nutrition (periods 3 and 4) were introduced after finalizing and analyzing the interviews from periods 1 and 2. The interview procedures are attached in Appendix 1.

The interviews were performed in four periods: Period one involved informal caregivers, period two involved care professionals, and period three involved PwD together with their caregivers and/or care professionals. In period four (February/March), interviews were performed with informal caregivers, some (N = 4) of whom had a migrant background. Interviews were conducted face-to-face, by telephone, or online.

### Data collection

Researchers and trained students executed the interviews. Students were studying Healthcare and Innovation in Healthcare. All students (N = 13) received instruction and training in conducting interviews with the target group and home visits. The participants were PwD or their caregivers and professionals involved in caring for PwD see Table 1. Most interviews were performed during the COVID-19 pandemic. Interviews are conducted from December 2021 until March 2023. The interviews lasted a maximum of 1 h and 15 min and were recorded with a voice recorder.

### Data analysis

Thematic analysis, as described by Braun and Clarke (Braun and Clarke 2006, 2019), was used. Thematic analysis can identify patterns within and between data on experiences, views, perspectives, behavior, and practices of participants. This method can help to understand what participants think, feel, and do (Clarke and Braun 2017). It was chosen to analyze all data combined since all interviews focused on the experiences and challenges of PwD and their caregivers during and around the night. The data collected from the PwD were treated similarly because these interviews were conducted in the presence and with the active participation of an informal caregiver. Inductive coding was used. Two researchers (first and second author) performed the first four steps within six interviews to determine an initial code list; with this code list, data from rounds 1, 2, and 3 were analyzed. Subsequently, a new topic list was developed based on the initial code list for the interviews in round 4. The rest of the dataset was reviewed and coded by the first and second authors separately. Researchers engaged in collaborative discussions throughout this process to resolve uncertainties and clarify interpretations. After coding with the initial code list, the first author examined the data again and added codes to identify the leads on which non-pharmacological interventions could support sleep quality. The Atlas.ti qualitative data software (version 23.2.3.27778) was used for data analysis (ATLAS.ti Scientific Software Development GmbH, 2023).

Based on the thematic analysis, the first, second, and third authors synthesized the results to visualize the findings. Finally, an inductive approach was taken to translate the qualitative findings into a custom experience flow. The experience flow approach brings forward the most important findings of a qualitative data set (McCarthy et al. 2016), better communicates the results with the target groups (Ayoola et al. 2016), and provides a structured mapping of the insights on a context-specific timeline (Brankaert 2016). This specific experience flow maps the experiences of PwD and their caregivers on a 24-h radial timeline, allowing us to view the experiences side by side and identify the most critical areas to focus on.

## Results

The study aimed to explore the issues, needs, and wishes of PwD and their caregivers concerning sleep at home, based on caregivers’ perspective. In addition, issues, needs, and wishes were visualized into an experience flow, which could contribute to identifying interventions for sleep support. Three main themes were identified, namely, a) challenges in maintaining time orientation and day/night routines, b) irregularities and concerns of informal caregivers at night, and c) environmental cues that either support or disturb the sleep of a person with dementia. The themes, including sub-themes, are shown in Appendix 2 and will be explained in detail in the following paragraphs. Table 2 shows the participants, their role, relationship to the person with dementia, and the profession of the formal caregivers.

**Table 2:** Overview of participants’ code, role, and relationship to the person with dementia

Code	Role	Relationship	Profession
D1	Informal caregiver and PwD	Partner	NA
D2	Informal caregiver and PwD	Partner	NA
D3	Informal caregiver	Granddaughter	NA
D4	Professional	NA	Daycare professional
D5	Informal caregiver	Partner	NA
D6	Professional	NA	Case manager
D7	Informal caregiver	Partner	NA
D8	Informal caregiver	Daughter	NA
D9	Informal caregiver	Partner	NA
D10	Informal caregiver	Partner	NA
D11	Informal caregiver	Daughter	NA
D12	Informal caregiver	Partner	NA
D13	Informal caregiver	Partner	NA
D14	Informal caregiver	Partner	NA
D15	Informal caregiver	Partner	NA
D16	Informal caregiver	Partner	NA
D17	Informal caregiver	Partner	NA
D18	Professional	NA	Case manager
D19	Professional	NA	Community nurse
D20	Professional	NA	Case manager
D21	Professional	NA	Community nurse
D22	Professional	NA	Community nurse
D23	Professional	NA	Case manager
D24	Professional	NA	Case manager
D25	Informal caregiver (double interview)	Daughters (N = 2)	NA
D26	Informal caregiver	Partner	NA
D27	Informal caregiver	Niece	NA
D28	Informal caregiver	Partner	NA

### Theme 1: challenges in maintaining time orientation and day/night routines

Some PwD experience issues with time orientation, which can disrupt the regular rhythm and affect sleep. A stable day/night rhythm with fixed activities can be helpful to keep structure during day and night. Furthermore, the number of activities during the day and evening is important for PwD. Physical discomfort may affect the night’s rest of PwD and activities during the day, affecting sleep. The interviews revealed that PwD, informal caregivers, and partners try to hold on to their existing routines as much as possible. This can, for example, be seen in the times they get up/wake up. At some point, there can be difficulties in motivating PwD to do tasks or specific activities, and then, day structure can be lost, which can impact the day–night rhythm and the sleep when a PwD does not want to go to bed. In addition, confusion occurs when, during the night, some PwD think that it is already morning and time to wake up. When a partner is present at night, they can point this out to reassure someone. There are also situations where the day structure is adapted to the (professional) care moments or the time someone has to go to daycare.

*"Yes, it depends. If we go to bed at half past eleven, we usually get up at half past seven. Even if the home care or cleaning lady comes and I know I have to get out of bed, sometimes I still prefer to stay in bed a bit longer." (Person with dementia – D1).*

It was also mentioned that naps are a regular part of the day structure; some participating professionals favored this, while other professionals would rather not see this because of the risk of disrupting the day–night cycle of a PwD. Daytime naps sometimes incur confusion as they make some individuals think it is morning again due to a lack of time awareness.

*“Of course, we'd rather not have a lot of sleeping during the day because then you have a chance that they will start reversing their day-night rhythm, and then at night, you really have a problem.” (Professional – D20).*

The sleep habits of PwD and their caregivers (when partners) depend on their past habits; some people have always been poor sleepers, and others have always been good sleepers. These habits do not change immediately when dementia occurs.

“*Sleeping is still going pretty well; actually, in my experience, she always sleeps well, but for a year now, she sometimes lies awake at night, though she has no intention to get out of bed yet.” (Informal caregiver – D7).*

Toilet visits of the PwD and informal caregivers were often mentioned as a reason for waking up at night. When a person with dementia returns to bed after going to the toilet, this is not a sleep disruption. However, a lack of place orientation or agitation can be seen as a sleep disruption.

In general, poor sleep of PwD leads to more fatigue during the day and sometimes to more naps. Naps may also lead to less sleep at night, leading sometimes to the reversal of the day–night rhythm. Also, poor sleep was mentioned as a result of reduced daytime functioning.

“*And that makes him, for instance, sit on the couch all day, well, and then he will doze off on the sofa, he will fall asleep because he is tired, of course, because he has been busy during the night. And so then you get a downward spiral very quickly. If something like that happens two days in a row to someone with dementia, it is very difficult to get out* *of it.” (Professional – D6).*

Furthermore, in some situations, PwD wake up early, eventually fall back asleep, and then sleep through until late, which disrupts their day–night rhythm as well.

Some participants pointed out trouble sleeping through the night of PwD. This was not necessarily perceived as a problem since people would not get out of bed. But it was a problem if, for example, agitation occurred. Finally, the challenge of falling asleep was mentioned and lying awake for a long time in both PwD and informal caregivers.

*"We always make sure we're both in bed by eleven. He can still stay awake for quite a while after that. The alarm is usually set for half past seven. Anything can happen in between." (Informal caregiver – D10).*

Agitation in the evening and night can be caused by major life events of PwD (e.g., death of a loved one), situations during the day (e.g., many visitors), and their emotions. This can cause problems such as falling asleep or sleeping through. Informal caregivers also mentioned that PwD sometimes start slapping, talking, or shouting at night. This may be related to re-experiencing reality or dreaming.

“*Because at night, he also cannot keep reality and non-reality apart.” (Informal caregiver – D12).*

Some PwD may become agitated if they fear their partner is no longer in bed; they need reassurance to calm down during the night when their partner takes on the task. When PwD leave the bed, sometimes there are problems finding the bedroom again, which causes stress. Wandering and ghosting were mentioned by caregivers as types of distress and potentially unsafe behavior.

“*And then I do notice that there is some scrambling. But then I do not react alertly, only to find after a half hour/3 quarter of an hour that she is no longer lying next to me and giving advice. And then I fish her up from the living room and put her back under only to find after half an hour/3 quarter of an hour that she is restless again and goes in, out, in, out, out, in, out. Sometimes she has an urge to go to the toilet, then it is logical that she wants to go out, but sometimes nothing else happens on the toilet, and she stays up until yes, what would it be yes 3 o'clock, 03.30 o'clock.” (Informal caregiver – D28).*

What caused the wandering remained unclear. In some cases, medication is used to deal with restlessness, but generally, medication is avoided. Instead, homeopathic remedies are considered for reassurance.

“*I have to say then that the example that touched me the most was when people are restless, they are often just missing a piece of safety and security.” (Professional – D18).*

Respondents indicate that everyday activities can make PwD tired, such as personal care or having breakfast. Some PwD then feel the need to rest for a while during the day. Contrastingly, it was mentioned that PwD who have enough stimuli and activities during the day (and no naps) are more tired in the evening, which can improve their sleep. On the other hand, some PwD fall asleep during moments without activities and stimuli, such as sitting in a chair (sometimes even with the TV on).

“*During the day, he sleeps quite a lot. I just can't do anything with it anymore. In the afternoon, I can still take him out on the bike for a while, but of course, he doesn't perform any activity himself then, of course.” (Informal caregiver – D13).*

Some physical problems cause PwD to become tired more quickly, or they may not be able to get out of bed on their own when they need to go to the toilet. This also disturbs the partner’s sleep because of the need for support.

*“Well, toilet visits were very frequent at night. But like I said, it's so hard for him to get out of bed.” (Informal caregiver – D12)*

Professionals indicated that PwD use more energy due to the presence of the dementia syndrome, which may cause them to need more sleep.

### Theme 2: irregularities and concerns of informal caregivers at night

The sleep and general well-being of an informal caregiver can be affected by the activities of a PwD during the night, which might cause worries about safety during the night or worries about the overall dementia situation. Convincing a person with dementia to stay active during the day or to go back to sleep can be a struggle for a caregiver.

When living together, the sleep of an informal caregiver can be disturbed when they are in distress or when the person with dementia is awake. Caregivers mentioned that informal caregivers are alert throughout the entire day and night, influencing their sleep quality.

*“The informal caregiver also has to deal with a disrupted day-night rhythm and that affects the carrying capacity of the informal caregiver.” (Professional – D6).*

According to several caregivers, sleep is crucial for informal caregivers since they have to provide care for PwD; to fulfill this task, adequate rest is essential. Professionals mention that when the nights of PwD or informal caregiver are disturbed, this might result in a transfer of PwD to a long-term care facility.

When the informal caregiver is not living with the person with dementia, there can be worries about what happens during both night and day. The impact of dementia can cause the caregivers to have worries and concerns, which may then affect their sleep quality. They worry about the next thing they have to deal with or what they have to do for the PwD, to be able to continue daily life. Participants mention that leaving someone alone could be unsafe, resulting in the caregiver feeling the person with dementia can no longer be on their own. In addition, during the night, the informal caregiver could fear the individual with dementia falling down the stairs or leaving the house without receiving an alarm.

*“You notice that you start to worry more and more when he tells you that he woke up at night and went outside and can't really tell you whether that was actually the case or whether it was just in his dreams. Yes, then you do feel like as a caregiver of, well, how long can he live here at home.” (Informal caregiver – D3).*

### Theme 3: environmental cues either support or disturb sleep

Participants mentioned that environmental factors can both support and hinder sleep. It is indicated that the environment can influence the behavior of a PwD, such as complete darkness can trigger the person to go back to sleep. Fresh air is indicated as a factor to improve sleep quality, based on the experiences of participants.

*"Otherwise, I can't sleep.* *Fresh air needs to come in because I need to be able to sleep." (Informal caregiver* *–* *D5)*

The environment can provide a sense of security for PwD, such as by having a nice blanket. Over time, however, it may become unsafe for someone with dementia to live by themselves, for example, when they start wandering at night, which requires adaptations of the environment to provide additional security.

*“Recently since two days ago, I put a chair in front of the bedroom door, and she's not able to realize that that's a chair that you can put away, and with that, I basically locked her in the bedroom. And I do that because she fell in the bathroom two weeks ago, and I don't want her to fall on the stairs and find her at the bottom of the stairs with some fractures or worse.” (Informal caregiver – D28).*

Several triggers are mentioned. A dark bedroom can be a signal that it is not yet time to get up, which can help a person with dementia to stay in bed and might fall asleep again. Another trigger mentioned is noise, which can make PwD restless and wake them up. Someone commented that earplugs are used to prevent this.

*“And these days, we also put in earplugs because he also suffers from overstimulation from a sound.” (Informal caregiver – D12*)

The night can be disturbed for PwD when they wake up without recognizing where they are. This can lead to the person looking for the home of their youth. Additionally, it occurs that PwD no longer know how to find their way into the present house, especially in the dark.

*“Well, sometimes he's awake, and then he calls my name, but then he doesn't know where he is. And then, yes then usually I do reassure him that he's just in his bed and that we're just here in this house.” (Informal caregiver – D13).*

### Translating the findings to an experience flow

**Fig. 1:**
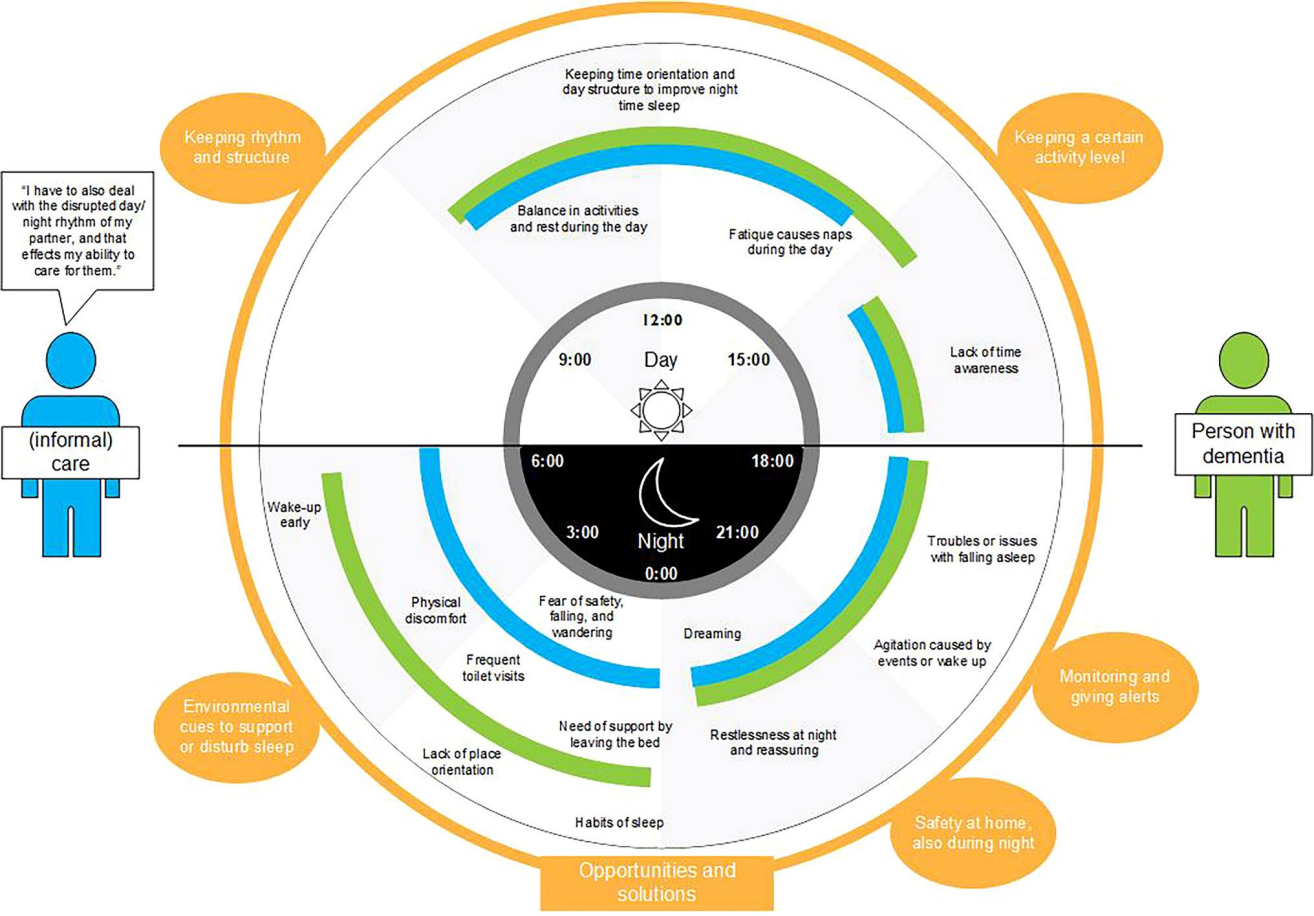
The experience flow of people with dementia and informal caregivers. The dark gray area indicates the nighttime. Blue = related to informal caregiver; green = related to the person with dementia; and orange = opportunities to position interventions

The experience flow was created to provide a more accessible and context-appropriate data analysis overview, translating the in-depth results of the thematic analysis. The experience flow highlights the difference in experiences for PwD and informal caregivers during the day and the night, which may influence sleep. By mapping this on a 24-h timeline, we can relate experiences to each other—these are experiences that might happen, and it does not intend to present a typical day. Furthermore, the experience flow presents opportunities in the outer line to provide additional support to improve sleep (for example, staying active through the day can improve sleep at night). This can be used to identify directions for caregivers, developers, and researchers to position future interventions and guide future research to support PwD and informal caregivers with sleep (Fig. 1).

The blue lines relate to the experiences of the (informal) caregiver mapped on a 24-h timeline. For example, during the day, supporting PwD in time orientation or day structure is challenging, as is finding a balance between activities and rest. For caregivers, the overview shows challenges with restlessness (e.g., realistic dreaming of PwD) and the worry and fear of the person with dementia. The overview summarizes the problem of informal caregivers using an exemplary quote: *“I also have to deal with the disrupted day/night rhythm of my partner, and that affects my ability to care for them.”*

The green lines relate to the experiences of the person with dementia on a 24-h timeline. During the day, PwD often struggle with time awareness and orientation, and they frequently experience fatigue. At night, they may face difficulties falling and staying asleep, along with challenges when awake, such as needing assistance to get out of bed or dealing with physical discomfort. The orange circle highlights opportunity categories identified during the interviews. For daytime, these opportunities include supporting a structured day and encouraging activity. For nighttime, we suggest (1) offering appropriate environmental cues, (2) opportunities for monitoring sleep and providing adequate feedback, and (3) general safety support.

## Discussion

This study aimed to explore the experiences that both PwD and caregivers have regarding sleep at home, to understand their challenges and needs, and to find direction on how to improve the day–night rhythm and overall sleep quality. It was not easy for participants to name needs and wishes. For instance, participants primarily discussed struggles with managing everything on their own and seldom brought up the need for support. The results of this qualitative study reveal the experiences of PwD at home, mostly according to their informal caregivers’ and the care professionals’ views. Three overarching themes were identified, which we will discuss further here and relate to possible support and interventions for PwD and informal caregivers.

### Positioning the themes and suggestions for non-pharmaceutical interventions

Our study provides insights into the needs of PwD and informal caregivers regarding sleep at home. The themes identified provide guidance to develop support regarding sleep. In this section, we discuss possible opportunities to translate the themes into possible (technological) interventions. This work is aided by a literature review, synthesizing sleep interventions (Huisman et al. 2022).

The first theme concerns maintaining time orientation and day structure to support sleep. The findings indicate that various issues and needs may arise. These include, such as going to bed too early, which can result in nocturnal awakenings. It appears that some PwD require assistance from formal or informal care to maintain this rhythm. The interview data reveal that the quantity and nature of daytime and evening activities appear to impact nocturnal behavior. An excessive number of activities can cause someone to fall asleep early in the evening due to fatigue, while too few activities can cause someone to become bored and fall asleep during the day. In their review, Ooms and Ju (2016) proposed that structured physical and social activities could improve sleep. Unfortunately, there is no standardized advice regarding daytime activity for PwD. Moreover, this is not even possible, since everybody responds differently. Rather, this should be approached more personally. This is also seen in the RCT of (Birkenhäger-Gillesse et al. 2020) in which a training program for informal caregivers is evaluated. Consistent with Ooms and Ju's (2016) review and the current research, it is imperative to consider the volume of activities for PwD. Thus, maintaining a consistent daily rhythm and balanced activities is crucial for managing sleep issues in PwD. Different support directions have already been explored. One approach is aromatherapy with essential oils to reduce sleep disturbances (Takeda et al. 2017). Another approach is giving reminders and cues to take action on appointments and needs by using for that purpose designed electronic calendar (e.g., (Kang et al. 2010; Topo et al. 2007). Another direction is to combine monitoring with giving cues to the person with dementia to activate (e.g., (Houben et al. 2022; Leyhe et al. 2020)). Our findings in this study suggest that we may be missing a focus for interventions to find a balance between daytime activities. Currently, caregivers guide day structure interventions. However, a system could be designed to align with the abilities of the person with dementia, suggesting suitable activities to support sleep in a person-centered manner.

The second theme highlights irregularities and concerns of informal caregivers, including safety concerns such as falls or disorientation, but also the entire situation they have to deal with. A primary concern is managing safety risks, such as potential falls and disorientation during nighttime wandering, which Ault et al. (2020) identify as a major contributor to caregiver burnout. Several support directions have been explored regarding this, for instance, improving safety at home by using monitoring with alarms via sensors (McKenzie et al. 2013; Rowe et al. 2009, 2010). Additionally, support for the care of a person with dementia includes reminding them to take their medication (Kort et al. 2019; McKenzie et al. 2013). The constant need for vigilance disrupts caregivers' sleep patterns, as they must remain alert to respond to unexpected situations, even during normal sleeping hours. Similar results of Lindeza et al. (2020) indicate that daily overload and PwD dependence can harm caregivers' health. Caregivers find that PwD dependence on daily living activities is challenging, and disease progression with night agitation and sleep issues worsens their sleep quality and causes fatigue (Lindeza et al. 2020). Therefore, it is crucial to understand the specific situation and challenges, as highlighted in the themes, that informal caregivers face regarding dementia. These factors should be incorporated into the support provided to them. There might be a need for enhancing the knowledge of the informal caregiver more directly regarding how to deal with dementia; this could be explored further concerning sleep of informal caregivers. Recent work of Houben et al. (2024) suggests that timely information and connecting caregivers to peers might provide sufficient support to cope with the daily challenges.

The third theme considers the physical environment, which can influence sleep. The results reveal that the lighting conditions are important for good sleep. This is in line with the study of Aarts et al. (2018) which shows that light affects sleep quality and daytime naps. Related to the physical environment, a sense of security is important because sometimes this is what a person needs to fall asleep. Despite other research indicating that the environment can elicit several problems, participants mentioned the environment as an influencing factor affecting PwD behavior less often. The previous studies show the influence of the (indoor) environment, for example, thermal comfort, lighting and contrast, and air quality (e.g., (Cerejeira et al. 2012; Holt Clemmensen et al. 2021; Tan et al. 2022), on PwD and how this can support several aspects, such as sleep, cognition, and activities of daily living. In general, our understanding of the influence of the environment on sleep of PwD needs to be further studied. The indoor environment should be considered when looking at supporting sleep of PwD and their caregivers (Hoof et al. 2010). The already explored support directions are about optimizing the sleep and living environment (e.g., (Radziszewski et al. 2016; Hoof et al. 2010), for example, by using suitable light conditions in every situation (Lieshout-van Dal 2023). Additionally, improving safety at home by using alarms or monitoring and supporting the person with dementia during the day (Kang et al. 2010; McKenzie et al. 2013; Topo et al. 2007; Tsolaki et al. 2015), as mentioned before. In this direction, we see researchers focusing studies on their expertise domain in research, e.g., isolating the impact of light in the paper by van Lieshout-van Dal (2023). Further interventions designed for practice should evaluate the environment more holistically and adjust only those factors that are problematic.

A study conducted by Curnow et al. (2021) identified twenty-four needs of PwD based on self-reports and reports by their informal caregivers. Our study confirms the necessity for daytime activities, physical health, behavior, and caregiving for others (Curnow et al. 2021). Additionally, this present study outlines and discusses potential support for improved sleep, recognizing sleep as another need, thereby adding to the body of knowledge.

### Limitations

The interpretation of the results provides leads for the development and implementation of non-pharmacological interventions to support sleep. Results describe the experience at this moment, but do not reveal the frequency with which experiences occur and do not say anything about the future. So, it should be left within the description of the PwD and the (informal) caregivers to judge whether a topic is important to focus on. The findings are highly context-specific, influenced by personal factors and living circumstances, this limits the transferability of the results. Recruitment challenges led to only four individuals with a migrant background, possibly due to hesitancy in seeking professional care. Insights from these interviews were similar to the other interviews. Although not many PwD participated in our study, this is comparable to other studies (Murphy et al. 2015). Including them in a study like this seems complicated because of their capabilities and frailty, which argues for research in which perspectives of caregivers are collected. In this study, the informal caregivers discussed their sleep and the sleep of PwD based on their perceptions and on behalf of a person with dementia. Because of the COVID-19 pandemic, many interviews were not conducted in person, which might have led to missing information, such as nonverbal cues, but sometime a video call was possible. Figure 1 visualizes the experiences concerning the sleep of informal caregivers and PwD. Due to the qualitative nature of this study, researchers’ biases could occur, and findings are specific to Dutch culture and healthcare system. Besides that, interviews are performed by different students and researchers, this may influence participants’ response.

## Conclusion

To conclude the results, three important themes are presented on which support can be focused. “Keeping time orientation and day structure” emerged as the main area of focus. Within this theme, it also appears relevant to look at caregiver support. In addition, this theme considers the indoor environment, which can support the problems experienced. The results of this study may offer guidance on developing or adjusting/non-pharmacological interventions for supporting the sleep of PwD and their informal caregivers. Appropriate support can significantly enhance the lives of PwD and their informal caregivers, thereby promoting aging-in-place. Results should be treated with caution due to the focus of the Dutch context.

## Data Availability

No datasets were generated or analyzed during the current study.

## Appendix 1

Interview topics

## Round 1

**Table:**

Topic	Question	Additional questions
Introduction	Introduce research and procedures	
General information	Can you tell something about yourself?	Living situation How long have you been an informal caregiver? Type and phase of dementia? How had the process been? How is the situation in and around the night? What changes occurred in sleep since the diagnosis of dementia?
Sleep of a person with dementia	Can you tell something about the sleep patterns of the person with dementia? (including going to bed and waking up)	How many hours of sleep? What time do you usually go to bed? How long does it take to fall asleep? At what time do you usually wake up in the morning? What causes you to wake up in the morning? Are their naps during the day? If so, how often/long? At what time of the day?
	Can you tell something about the sleep habits of the person with dementia?	Before sleep Going to bed Waking up
	What do you experience in the person with dementia during the night regarding sleep?	Is there restlessness during the night? Wandering around? Dreaming? Toilet use? Frequent waking up? Waking up and not being able to get back to sleep? Other experiences/ reasons?
	How do any sleep problems affect a person with dementia?	
	What does the person with dementia do to have good sleep, or what has been tried in the past?	
	What does the person with dementia need to have a good night’s sleep?	Medication Sleep habits Certain rituals? Other wishes/needs? Support from others? Other support? Is there support already?
Impact on informal caregiver	How do any sleep problems affect you?	Impact on daily living
	What do you need to keep the burden acceptable?	
Wrap up	Do you want to add something to this conversation?	

## Round 2

**Table:**

Topic	Question	Additional questions
Introduction	Introduce research and procedures	
Care professional	What is your current position?	Work experience
	Can you tell me something about the patient population you have experience with?	Which patient population is common?
Dementia	Can you tell a bit more about the patient group of people with dementia in-home care?	Type of dementia Phase of dementia Living situation Informal caregiver
Sleep	We focus on the night situation of people with dementia	
	Can you tell what you see regarding sleep patterns in people with dementia in the home setting?	Common problems Ritual going to bed Time to bed Average hours of sleep Sleep quality Fall asleep/sleep through Night rest Restlessness? Wandering? Toilet visits? Unable to sleep/wake up
	What kind of problems do you identify in people with dementia in and around the night in the home setting?	Sleep problems Disturbed day and night rhythm?
	What are things the person with dementia does to get a good night’s sleep?	Medication Sleep habits Certain rituals? Assistive devices?
	What do you think the person with dementia needs to have a better night’s sleep?	
Informal caregiver	What kind of problems do you identify in and around the night in informal caregivers of people with dementia?	Overload/overburden? Stress? Fatigue?
	What might help the informal caregivers with these problems?	
Wrap up	Are there things you would like to say that might add value to this research?	
	Do you have any questions?	

## Round 3

**Table:**

Main topic	Subtopic	Additional
General	Gender	
	Age	
	Background	Non-Western Western 1st or 2nd generation
	Education level	
	Living situation	
	Residence/district	
Sleep	How many hours of sleep?	
	Sleep problems?	Medication? Difficulties falling asleep? Difficulty sleeping through? Waking up early? Not waking up well-rested? Feeling sleepy during the day?
Factors which can influence sleep	Physical fatigue/activities during the day à sleep better	Sport and exercises during the day Inactivity during the day Exercise just (< 3 h) before bedtime Relaxation before bedtime Excessive cognitive and emotional activity just before bedtime Frequency and duration of daytime naps
	Food intake and sleep	Eating solid food just (< 3 h) before bedtime Using caffeine just (< 3 h) before bedtime Using alcohol just (< 3 h) before bedtime
	Stimuli just before bedtime	Light from TV/smartphone, tablet, etc. Relaxation before bed (think of walking, hot bath, etc.)
	Bedroom	Fresh air/ventilation Dark Quiet bedroom Temperature cooler than the living room Humidity (not too dry) Avoid noise (snoring, etc.)—possibly earplugs
	Sleep rhythm (time going to bed—waking up)	Regular sleep rhythm Staying up longer Frequent sleepovers Naps during the day
	Not falling asleep (after 15 min not asleep)	Waiting Reading, preferably in another room Listening to, e.g., music, preferably in another room Go to sleep later—try to get up according to the regular rhythm
	Presence of restlessness	Restlessness causes poor sleep Causes of restlessness
	Sleep debt	Build-up of sleep debt: the longer awake, the higher the need for sleep Decrease in sleep debt
	Biological clock	Morning people vs Evening people Short sleepers (average 6 h) vs. long sleepers (average 10 h) Menopausal women—changes in sleep (hormonal/nocturnal hot flashes)
	Melatonin	Hormone indicates optimal time to sleep
	Lifestyle	Overweight (BMI > 28)
Wrap up	Anything to add?	

## Round 4

**Table:**

Main topic	Subtopic	Additional
Introduction	Explanation of research and procedures	
General information	Relationship	
	Demographic information	Age Etc.
	Living situation	
	Other limitations/diseases/disorders	
	Personal characteristics	
Living with dementia	What does daily life look like?	Day structure Day activities Household tasks Nutrition Support from others
Sleep/night	How does the night go for you and the person with dementia?	Bed rhythmical/rhythm/time Getting up Sleep medication Problems while sleeping Changes in sleep pattern Sleeping/naps during the day Sleep quality of the person with dementia Physical environment bedroom Support
Others	Barriers	
	Use of technology	
Wrap up	Additional points	

## Appendix 2

See Table

**Table 3:** Overview of themes, sub-themes, and quotes based on the interviews

	Theme	Sub-themes	N	Example quotes to illustrate sub-theme (translated from Dutch)
1	Challenges in maintaining time orientation and day/night routines	Time orientation and structure during the day	28	“You do that partly by advising that they take a rest at noon so they have more energy left but also that they provide distractions in the evening.” (Professional)
				“Of course, we'd rather not have a lot of sleeping during the day because then you have a chance that they will start reversing their day-night rhythm, and then at night, you really have a problem.” (Professional)
				"Yes, it depends. If we go to bed at half past eleven, we usually get up at half past seven. Even if the home care or cleaning lady comes and I know I have to get out of bed, sometimes I still prefer to stay in bed a bit longer." (Person with dementia)
		Disruption of night's rest	25	“Sleeping is still going pretty well; actually, in my experience, she always sleeps well, but for a year now, she sometimes lies awake at night, though she has no intention to get out of bed yet.” (Informal Caregiver)
				“And that makes him, for instance, sit on the couch all day, well, and then he will doze off on the sofa, he will fall asleep because he is tired, of course, because he has been busy during the night. And so then you get a downward spiral very quickly. If something like that happens two days in a row to someone with dementia, it is very difficult to get out of it.” (Professional)
				"We always make sure we're both in bed by eleven. He can still stay awake for quite a while after that. The alarm is usually set for half past seven. Anything can happen in between." (Informal Caregiver)
		Causes of distress for a person with dementia during day- and nighttime	21	“That gentleman I was just talking about, he starts talking. So he wakes up his wife. And then he starts talking about something, yes, whatever is on his mind at that moment.” (Professional)
				“Because at night, he also cannot keep reality and non-reality apart.” (Informal Caregiver)
				“And then I do notice that there is some scrambling. But then I do not react alertly, only to find after a half hour/3 quarter of an hour that she is no longer lying next to me and giving advice. And then I fish her up from the living room and put her back under only to find after half an hour/3 quarter of an hour that she is restless again and goes in, out, in, out, out, in, out. Sometimes she has an urge to go to the toilet, then it is logical that she wants to go out, but sometimes nothing else happens on the toilet, and she stays up until yes, what would it be yes 3 o'clock, 03.30 o'clock.” (Informal Caregiver)
				“I have to say then that the example that touched me the most was when people are restless, they are often just missing a piece of safety and security.” (Professional)
		Balance in activities and rest during the day	15	“During the day, he sleeps quite a lot. I just can't do anything with it anymore. In the afternoon, I can still take him out on the bike for a while, but of course, he doesn't perform any activity himself then, of course.” (Informal Caregiver)
		Physical discomforts that limit the independence of a person with dementia	7	“Well, toilet visits were very frequent at night. But like I said, it's so hard for him to get out of bed.” (Informal Caregiver)
2	Irregularities and concerns of informal caregivers at night	Disturbance of the nights’ rest of an informal caregiver	19	“The informal caregiver also has to deal with a disrupted day-night rhythm and that affects the carrying capacity of the informal caregiver.” (Professional)
		Worries of an informal caregiver of a person with dementia in and about the night	11	“You notice that you start to worry more and more when he tells you that he woke up at night and went outside and can't really tell you whether that was actually the case or whether it was just in his dreams. Yes, then you do feel like as a caregiver of, well, how long can he live here at home.” (Informal Caregiver)
3	Environmental cues either support or disturb the sleep of a person with dementia	Influence of the environment on sleep and safety	14	“Recently since two days ago, I put a chair in front of the bedroom door, and she's not able to realize that that's a chair that you can put away, and with that, I basically locked her in the bedroom. And I do that because she fell in the bathroom two weeks ago, and I don't want her to fall on the stairs and find her at the bottom of the stairs with some fractures or worse.” (Informal Caregiver)
				"Otherwise, I can't sleep. Fresh air needs to come in because I need to be able to sleep." (Informal Caregiver)
		Triggers that may disturb the sleep of a person with dementia	12	“And these days, we also put in earplugs because he also suffers from overstimulation from a sound.” (Informal Caregiver)
		Lack of place orientation of a person with dementia in the home environment	9	“Well, sometimes he's awake, and then he calls my name, but then he doesn't know where he is. And then, yes then usually I do reassure him that he's just in his bed and that we're just here in this house.” (Informal Caregiver)

3
